# Macromolecular changes in spinal cord white matter characterize whiplash outcome at 1-year post motor vehicle collision

**DOI:** 10.1038/s41598-020-79190-5

**Published:** 2020-12-17

**Authors:** Mark A. Hoggarth, James M. Elliott, Zachary A. Smith, Monica Paliwal, Mary J. Kwasny, Marie Wasielewski, Kenneth A. Weber, Todd B. Parrish

**Affiliations:** 1 Department of Biomedical Engineering, McCormick School of Engineering, Northwestern University, Evanston, IL USA; 2 Department of Physical Therapy and Human Movement Sciences, Feinberg School of Medicine, Northwestern University, Chicago, IL USA; 3 Northern Sydney Local Health District, The Kolling Research Institute and The Faculty of Health Sciences, The University of Sydney, St. Leonards, NSW Australia; 4 Department of Neurological Surgery, University of Oklahoma Health Sciences Center, Oklahoma City, OK USA; 5 Department of Preventive Medicine, Feinberg School of Medicine, Northwestern University, Chicago, IL USA; 6 Systems Neuroscience and Pain Lab, Department of Anesthesiology, Perioperative and Pain Medicine, Stanford University, Palo Alto, CA USA; 7 Department of Radiology, Northwestern University, Chicago, IL USA

**Keywords:** Magnetic resonance imaging, Diagnostic markers, Spinal cord diseases

## Abstract

Each year, whiplash injuries from motor vehicle collisions (MVC) affect millions worldwide, with no strong evidence of pathology. While the majority recover soon after the injury, the same is not true for roughly 20% reporting higher levels of pain and distress, without diagnostic options. This study used magnetization transfer (MT) imaging to quantify white matter integrity in 78 subjects with varying levels of pain, 1 year after MVC. MT images of the cervical spinal cord were collected parallel to the intervertebral disks. MT ratios (MTR) were calculated in select white matter tracts along with MTR homogeneity (MTR*h*) at each level. Significant differences were observed between clinical outcome groups in the left and right spinothalamic tracts (*p* = 0.003 and 0.020) and MTR*h* (*p* = 0.009*).* MTR*h* was elevated in females with poor recovery versus females reporting recovery (*p* < 0.001) or milder symptoms (*p* < 0.001), and in males reporting recovery (*p* = 0.007) or no recovery (*p* < 0.001). There was a significant interaction between recovery status and sex (*p* = 0.015). MT imaging identified tract specific and regional changes in white matter integrity suggesting potential insults to the cord. Additionally, significant MTR*h* differences between sexes were observed, characterizing the heterogeneity of whiplash recovery and worse outcomes in females.

## Introduction

Whiplash injuries are the most common outcome from non-catastrophic motor vehicle collisions (MVC), affecting nearly four million people in the United States each year^1,2^. The collision related mechanics of the event give rise to the term, whiplash, where the subsequent signs and symptoms are often complex and known clinically as whiplash associated disorders (WAD)^3–5^. While nearly 50% of those injured should expect rapid recovery, the remainder will continue to report symptoms in the longer term. Nearly a quarter of those injured (up to one million) will present with a wide variety of signs and symptoms characterized by neck pain, headache, widespread sensory hypersensitivity, changes in motor performance, cognitive interference, changes in muscle composition, and psychopathology^6–10^. Effective treatments for WAD are limited, which reflect the paucity of research identifying potential underlying mechanisms of and causal inferences for persistent WAD^11–13^.

Given the pathomechanics of a typical MVC (e.g. rear-end, side, or frontal impacts), a subtle insult to the cervical spinal cord is feasible^14–16^, but such an insult is likely radiologically occult with conventional imaging. Clinical observations and case-series research reports^9,16^ of heightened reflexes and corticomotor signs (e.g. Babinksi Reflex, Clonus) suggest injury involving the cord in some people^17,18^ and this may feature in those with narrower spinal canals^19^. Magnetization transfer (MT) imaging, an advanced MRI method, could provide quantification of a discrete cord insult following MVC. MT imaging is performed by combining two scans, one having added contrast from protons bound to macromolecules, such as myelin. Comparison of a standard image to a bound-proton saturated image allows for indirect measure of bound (macromolecular) protons^20^. MT imaging is sensitive to demyelination of the spinal cord pathways in those with incomplete spinal cord injury (iSCI)^9,21^ and multiple sclerosis^22,23^.

It is plausible MT imaging could provide an early and accurate quantification of an otherwise radiologically occult insult involving the spinal cord, which could (1) prove prognostic, (2) inform clinical trials, and (3) provide improved management schemas for patients left with little diagnostic options to explain their persistent pain and disability. The purpose of this cross-sectional study was to investigate whether MT imaging was able to identify those participants injured from an MVC whose persistent pain may be consistent with an insult to cervical spinal cord white matter, in general, and pathways, specifically.

## Results

A total of 76 participants were analyzed for this study: 30 (39%) reporting recovery; 32 (42%) reporting continuing mild symptoms; and 14 (18%) reporting continued severe symptoms. Demographics for each group are provided In Table 1. There was no significant difference in age or BMI between clinical outcome groups. Initial NDI percentage scores for the mild (39.8%) and severe (49.8) groups were not significantly different from each other (*p* = 0.303), but were both higher than the recovered group (25.2%, *p* < 0.001 for both cases). At 1 year, NDI scores (recovered = 5.53%, mild = 19.2%, and severe = 29.7%) were significantly different between all groups (*p* < 0.001 for all cases). The analysis was limited to images acquired at C2/3, C3/4, C4/5, and C5/6 due low number of scans with sufficient imaging quality at C6/7 and C7/T1. The number of scans analyzed at each level are provided in supplementary Table S1.

**Table 1:** Descriptive statistics from study participants, grouped by clinical outcome.

	Recovered		Mild		Severe	
Number of participants	30		32		14	
Age (year)	31.7	(10.10)	35.9	(12.50)	36.1	(11.30)
Body mass index (kg/m^2^)	23.9	(3.13)	24.2	(4.20)	26.7	(4.72)
%Female	57%		88%		79%	
Initial NDI (%)	25.2*	(13.70)	39.8	(14.20)	49.8	(13.90)
NDI at year 1 (%)	5.53*	(6.40)	19.2*	(10.20)	29.7*	(13.60)

Outcome was determined by Neck Disability Index (NDI) percentage scores. *p < 0.001.

Significant differences were observed between the 3 clinical outcome groups when comparing means of left and right spinothalamic tracts (*p* = 0.003 and 0.02 respectively) and MTR*h* (*p* = 0.009). All ANOVA results with clinical outcome as a factor are shown in Table 2. Post hoc analysis, shown in Table 3, showed significant group MTR*h* differences between the recovered and severe group (*p* = 0.011), and between the mild and severe groups (*p* = 0.024). Similarly, in the left spinothalamic tract, significant differences were observed between the mean MTR of the severe and recovered (*p* = 0.003) and mild (*p* = 0.010) groups. The right spinothalamic tract MTR was significantly different between the severe and mild groups only (*p* = 0.017).

**Table 2:** ANOVA results between clinical group white matter tract magnetization transfer ratio (MTR) and MTR homogeneity (MTR*h*) means using clinical outcome as a factor.

Tract	Sum of squares	df	Mean square	F	*p*
L CST	70.443	2	35.221	1.333	0.266
R CST	56.059	2	28.029	1.047	0.353
L CF	67.636	2	33.818	1.87	0.156
R CF	97.07	2	48.535	2.026	0.134
L GF	10.341	2	5.17	0.299	0.742
R GF	70.128	2	35.064	1.914	0.150
L SPTH	373.513	2	186.757	6.122	0.003
R SPTH	160.653	2	80.327	3.998	0.020
MTR*h*	0.021	2	0.01	4.785	0.009

White matter tracts are the left and right corticospinal tracts (L and R CST), cuneate fasciculus (L and R CF), gracile fasciculus (L and R GF), and the combined spinothalamic and spinoreticular tracts (L and R SPTH).

**Table 3:** Post-hoc comparisons with Bonferroni adjusted *p* values of magnetization transfer ratio (MTR) from white matter tracts and MTR homogeneity (MTR*h*) between clinical outcome groups. White matter tracts above are the left and right combined spinothalamic and spinoreticular tracts (L and R SPTH).

	Comparison			Mean difference	95% CI for mean difference				
					Lower	Upper	SE	t	Adj. *p*
MTR*h*	Recovered	:	Mild	− 0.001	− 0.017	0.015	0.007	− 0.206	1.000
		:	Severe	− 0.024	− 0.043	− 0.005	0.008	− 2.929	0.011
	Mild	:	Severe	− 0.022	− 0.042	− 0.003	0.008	− 2.678	0.024
L SPTH	Recovered	:	Mild	− 0.272	− 2.169	1.625	0.804	− 0.338	1.000
		:	Severe	− 3.207	− 5.467	− 0.946	0.958	− 3.345	0.003
	Mild	:	Severe	− 2.935	− 5.261	− 0.608	0.987	− 2.975	0.010
R SPTH	Recovered	:	Mild	− 1.089	− 2.628	0.451	0.653	− 1.668	0.290
		:	Severe	1.141	− 0.693	2.976	0.778	1.467	0.431
	Mild	:	Severe	2.23	0.342	4.118	0.801	2.786	0.017

In the second stage of analysis, with the inclusion of sex along with status as a factor in the ANOVA model, as shown in Table 4, significant differences were observed in the left spinothalamic tract (*p* = 0.002) and MTR*h* (*p* < 0.001) means, but not for the right spinothalamic tracts (*p* = 0.052). In post hoc analysis of MTR*h*, significance was found between the severe outcome group of females versus recovered females (*p* < 0.001), females with mild outcome (*p* < 0.001), males who recovered (*p* = 0.007), and males with severe outcome (*p* < 0.001). Post hoc analysis of the left spinothalamic tract showed significant group differences between females in the severe group versus recovered females (*p* < 0.001), females with mild outcome (*p* = 0.024), and males with mild outcome (*p* = 0.027). All post hoc comparisons for the left spinothalamic tract MTR and MTR*h* metric are shown in supplementary Table S2.

**Table 4:** ANOVA results between clinical group white matter tract magnetization transfer ratio (MTR) homogeneity and MTR homogeneity (MTR*h*) means using status and sex as factors.

	Cases	Sum of squares	df	Mean square	F	*p*
L SPTH	Status	160.654	2	80.327	2.754	0.066
	Sex	20.216	1	20.216	0.693	0.406
	Status * Sex	387.84	2	193.92	6.648	0.002
R SPTH	Status	52.806	2	26.403	1.339	0.264
	Sex	0.422	1	0.422	0.021	0.884
	Status * Sex	118.05	2	59.025	2.993	0.052
MTR*h*	Status	0.003	2	0.001	0.685	0.505
	Sex	0.005	1	0.005	2.606	0.108
	Status * Sex	0.04	2	0.02	9.902	< 0.001

White matter tracts above are the left and right combined spinothalamic and spinoreticular tracts (L and R SPTH).

**Figure 1:**
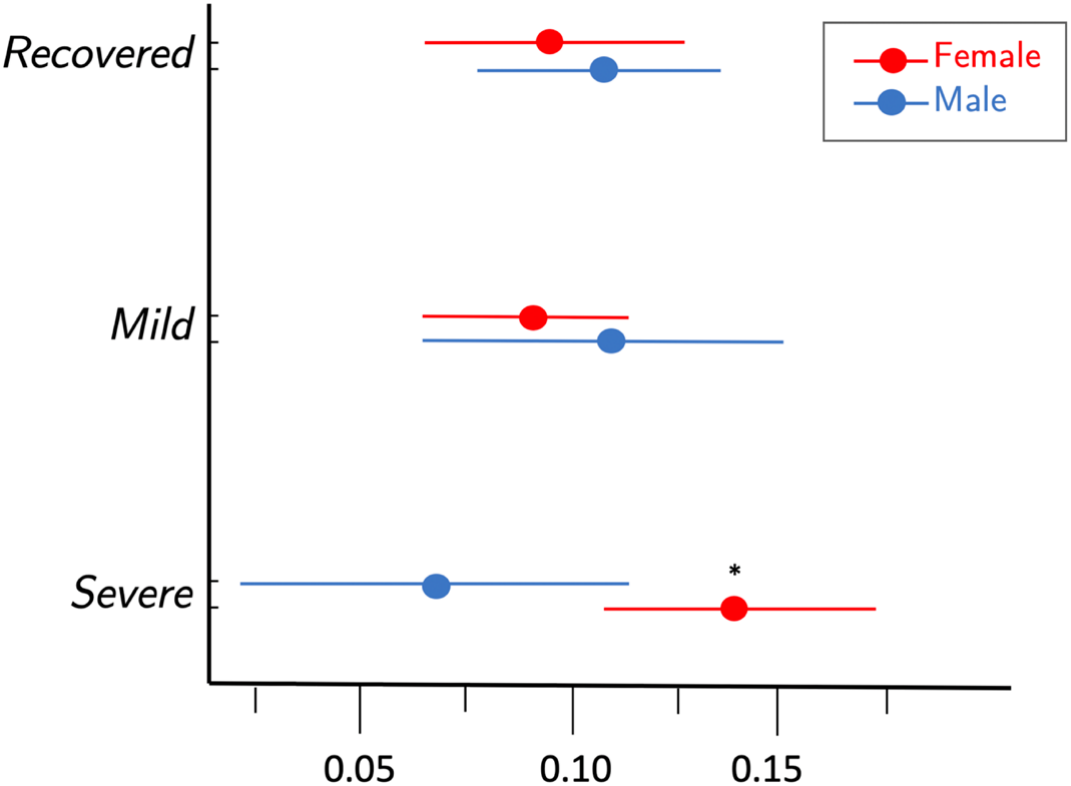
Least square mean homogeneity index of the magnetization transfer ratio (MTR*h*) with 95% confidence intervals from linear mixed model for females and males by recovery status, adjusting for tract (of lateral corticospinal, spinothalamic, spinoreticular, and gracile and cuneate fascicle), intervertebral disk (C2/3, C3/4, C4/5, C5/6), side, BMI, and age. *p < 0.05 compared to females in recovered and mild groups and males in the severe group.

In the third stage of the analysis, with regard to MTR*h* values, there was a significant interaction between recovery status and sex (*p* = 0.015) in the mixed linear model. As shown in Table 5 and Fig. 1, least square means for the female and male recovered groups were 0.094 and 0.111; mild groups were 0.087 and 0.112; severe were 0.140 and 0.067, respectively. Least square mean MTR*h* values for females with severe clinical outcome were significantly greater from the recovered group (*p* = 0.023), mild group (*p* = 0.005), and males with severe outcome (0.010). Least mean square differences are given in Table 6. In regard to MTR values for the large white matter tracts in the linear mixed model, no significant differences were observed. Least square means for each tract, as reported in Table 7, were combined across multiple levels, and separated by sex. As shown in Table 8, significant correlations were not observed between MTR and NDI, nor MTR*h* and NDI, save for the combined left spinothalamic and spinoreticular tracts at levels C3/4 and C4/5 with weak positive relationships (r = 0.26, *p* = 0.037; r = 0.29, *p* = 0.023), and the MTR*h* metric at C3/4 with a weak positive relationship (r = 0.27, *p* = 0.034).

**Table 5:** Least square mean magnetization transfer ratio homogeneity from linear mixed model for clinical outcome groups by sex, including imputed data.

Status	Sex	N	Estimated	95% confidence interval	
			Mean	Lower	Upper
Recovered	Female	17	0.094	0.064	0.123
Recovered	Male	13	0.111	0.082	0.139
Mild	Female	28	0.087	0.063	0.110
Mild	Male	4	0.112	0.067	0.157
Severe	Female	11	0.14	0.106	0.174
Severe	Male	3	0.067	0.017	0.116

**Table 6:** Differences in least square mean magnetization transfer ratio homogeneity from general linear mixed model when controlling for clinical outcome and sex, including imputed data.

Comparison group			Within	Estimated difference	95% confidence interval		*p*
					Lower	Upper	
Recovered	vs	Mild	Males	− 0.001	− 0.047	0.05	0.953
Recovered	vs	Severe	Males	− 0.044	− 0.096	0.008	0.095
Mild	vs	Severe	Males	0.046	− 0.014	0.105	0.131
Recovered	vs	Mild	Females	0.007	− 0.034	0.02	0.626
Recovered	vs	Severe	Females	− 0.046	− 0.006	− 0.087	0.023
Mild	vs	Severe	Females	− 0.053	− 0.09	− 0.017	0.004
Men	vs	Female	Recovered	− 0.017	− 0.05	0.016	0.303
Men	vs	Female	Mild	− 0.025	− 0.069	0.018	0.248
Men	vs	Female	Severe	− 0.073	− 0.017	− 0.129	0.011

**Table 7:** Least square mean magnetization transfer ratio values from linear mixed model of each tract for clinical outcome groups by sex, including imputed data.

Tract	Recovered		Mild		Severe	
	Female	Male	Female	Male	Female	Male
L CST	38.7	40.9	39.9	36.0	40.7	41.7
R CST	39.9	38.4	40.2	37.0	37.7	40.7
L CF	39.5	39.7	40.3	38.2	40.9	41.9
R CF	39.6	38.9	40.7	38.6	38.4	40.5
L GF	40.8	39.7	41.0	39.8	41.0	40.8
R GF	40.5	39.7	41.1	39.7	39.8	39.8
L SPTH	38.1	40.8	40.0	37.9	43.4	40.0
R SPTH	41.3	39.0	41.4	40.3	38.8	41.0

White matter tracts above are the left and right corticospinal tracts (L and R CST), cuneate fasciculus (L and R CF), gracile fasciculus (L and R GF), and the combined spinothalamic and spinoreticular tracts (L and R SPTH).

**Table 8:** Pearson correlations (with *p* value) between Neck Disability Index and imaging measures at each cervical level.

	C2/3		C3/4		C4/5		C5/6	
L CST	0.18	(0.157)	− 0.10	(0.441)	0.22	(0.072)	0.26	(0.072)
R CST	− 0.13	(0.304)	− 0.06	(0.622)	− 0.04	(0.778)	− 0.16	(0.271)
L CF	0.15	(0.238)	− 0.01	(0.959)	0.23	(0.067)	0.22	(0.108)
R CF	− 0.05	(0.688)	− 0.12	(0.343)	0.09	(0.507)	0.03	(0.856)
L GF	− 0.03	(0.816)	0.09	(0.475)	0.18	(0.163)	0.09	(0.594)
R GF	0.02	(0.888)	− 0.08	(0.522)	0.13	(0.340)	0.05	(0.745)
L SPTH	0.18	(0.155)	0.26	(0.037)	0.29	(0.023)	0.12	(0.397)
R SPTH	− 0.15	(0.228)	− 0.21	(0.086)	0.01	(0.945)	0.05	(0.727)
MTR*h*	0.20	(0.180)	0.27	(0.034)	0.18	(0.187)	0.22	(0.126)

White matter tracts above are the left and right corticospinal tracts (L and R CST), cuneate fasciculus (L and R CF), gracile fasciculus (L and R GF), and the combined spinothalamic and spinoreticular tracts (L and R SPTH).

## Discussion

This investigation of participants at 1-year after an MVC illustrates the potential utility of MT imaging of the spinal cord to identify white matter tract specific and regional changes in cord integrity. Significant differences in MTR and MTR*h* were uniquely observed in the severe whiplash female group compared to the other clinical outcomes and sex. These findings support the wider literature and clinical observations of potential subtle insults to the spinal cord in those with severe WAD^9,16^. As shown in Table 7, tract-specific mean MTR values by group were similar in magnitude. Shown in Fig. 1, the group of female participants with a worse clinical outcome had generally increased MTR*h* (i.e. loss of homogenous MT) when compared to their male and female counterparts in the other recovery groups. Although significant as a group, the total number of subjects in the severe group was small (N = 14), and consistent with the wider-body of literature, a majority were female (79%).While it is unknown what sex-related physiological differences (e.g. musculoskeletal structure and hormonal makeup), will have on imaging of the spinal cord after trauma, these sex-dependent findings warrant further longitudinal and cross-sectional studies evaluating MT imaging techniques in large groups of symptomatic and asymptomatic participants of varying ages.

It is recognized that a subtle, but focal, insult to the cord will likely not create a large disturbance in MT signal. While studies have shown decreased MTR evident in corticospinal tracts of those with known motor iSCI, these lesions are notably large when compared to the potentially subtle injury occurring in those studied in this work^21,24^. Accordingly, we have proposed the MTR*h*, a participant-normalized metric, as a method to identify subtle changes in the distribution of the MT signal in the cord that may be related to spinal cord white matter demyelination. MTR*h* presents a method of analyzing the spinal cord without a priori hypotheses of suspected areas of injury, and with future work for validation and reliability could be used for identifying specific areas of subtle cord demyelination.

Given the group differences in MTR*h*, but lack of significant correlation with NDI, this data supports the widely noted observations of the heterogeneity of WAD recovery. Specifically, changes in MTR*h* identifying altered white matter myelination illustrate a mild cord insult may feature in some (not all) with poor outcome following an MVC. It also follows that potential biological injuries can have grossly different clinical outcomes, representing complex interactions between tissue and stress-based interactions, underpinning the importance of consistently using a core set of outcome measures for whiplash^25,26^.

A limitation of this study is that the number of useable images, with sufficient white and grey matter contrast and lacking any artifacts, decreased from the initial C2/3 interspace to the most inferior at C7/T1. Contrast between white and grey matter is required for accurate registration to the template and then for delineation of white matter tracts for measures of MTR in the cord. The imaging protocol gathered slices sequentially to minimize the effects of motion during the acquisition of each slice. Images from the lower cervical spine have lower signal to noise due to poor receive coil coverage and therefore more prone to motion artifacts from pulsatile flow, breathing, and, when considering the participants in this study reported neck pain, participant motion in the scanner. Furthermore, the imaging protocol adopted for this work relied on a lateral phase encoding direction to minimize artifacts from anterior and posterior pulsatile flow of the cord, however, when images were acquired at lower levels there is more tissue in the lateral direction due to the shoulders; this could lead to aliasing artifacts. It should be noted that the MT signal contrast represents a broad spectrum of macromolecule signals from myelin, edema, inflammatory response, and other proteins. In this paper we have interpreted these signal changes as a result of changes in myelination, but this could be confounded by the presence of edema and inflammatory events as a result of injury^22,27,28^. Future work, looking at participant-specific repeated measures of tract specific MTR and MTR*h* reliability is warranted and underway.

This work supports the need for continued high-resolution imaging of the human spinal cord in those with whiplash injuries^29,30^. These findings support the need for future investigations, utilizing tools such as MT imaging, into the potential for focal insults to the cervical spinal cord in a discrete number of patients with WAD who present with more complex signs and symptoms where little, if any, diagnostic (or therapeutic) options exist^31,32^.

## Methods

### Participants

Participants were selected from a prospective parent study of 97 injured participants presenting to an urban emergency medicine department (ED) with Level 1 trauma distinction following an MVC (ClinicalTrials.gov Identifier: NCT02157038). In accordance with the Declaration of Helsinki, all institutional and governmental regulations were followed, with ethical approval obtained from the Institutional Review Board at Northwestern University. All participants provided informed written consent prior to data collection, and all personal identifying information was removed from the datasets.

Participants within the Quebec Task Force (QTF) classification category of WAD Grade II (neck tenderness to palpation, movement restriction with no radicular symptoms) were recruited directly from the ED^5^ as this represents the most common category where an acknowledged heterogeneity in the clinical presentation exists^33^. No participant was diagnosed with an injury involving the spinal cord or other soft-tissues of the cervical spine. Exclusion criteria were younger than 18 or older than 65 years of age, one or more previous MVCs in their lifetime, treatment for neck pain disorders in the past 10 years, any nervous system disorder (e.g. stroke, Parkinson's), metabolic system disorder (e.g. diabetes), bony fracture of the cervical spine, hemorrhage, or those who, by standard ED protocols were deemed to be at risk for multi-system trauma. All participants were followed and assessed by NDI and imaging at < 1-week, 2-weeks, 3-months, and 1-year post injury as part of the prospective study.

Of the 97 participants who enrolled in and completed the longitudinal study, 78 completed all 4-time points. The Neck Disability Index (NDI) was administered to all participants at each assessment. This study analyzed MT images (N = 78) of the cervical spinal cord taken at the 1-year post-MVC time point. Participants in this study were classified based on NDI % scores at 1-year post-MVC as recovered (NDI < 10%), mild (NDI 10–28%), or severe (NDI > 28%)^34^. Age (years), sex, and BMI (Kg/m^2^) were captured using a standardized self-report form.

### Magnetization transfer imaging protocol

MRI images were collected parallel to each of the C2/3, C3/4, C4/5, C5/6, C6/7, and C7/T1 intervertebral disks (multi-slice multi-angle) using the Multiple-Echo Data Image Combination (MEDIC) sequence^35^ on a Siemens (Erlangen, Germany) 3 T Prisma scanner. The MEDIC sequence combines multiple echoes to increase signal-to-noise and reduce pulsatile artifacts, without increasing scan time or specific absorption rate. The 2D MEDIC sequence parameters were: TR/TE_eff_/Flip angle of 300 ms/17 ms/40° combining four echoes 7.68, 13.75, 19.83, 26.12 ms, matrix size of 384 × 384, field of view (FOV) of 180 × 180 mm, left-to-right phase-encoding direction, in-plane resolution of 0.5 mm, and a slice thickness of 4 mm. Parallel imaging, via generalized auto calibrating partial parallel acquisition (GRAPPA), with an acceleration of 2, was used to reduce scan time and motion artifacts^36^. A sequential acquisition scheme was utilized by acquiring a single level completely and then moving on to the next level to minimize motion effects. In order to detect the presence of macromolecules using the magnetization transfer effect, two nearly identical scans are required, one with an MT off-resonance saturation pulse and one without. The MT pulse was applied 1.5 kHz off-resonance with a large (540°) flip angle and long duration (10 ms) to saturate the bound water pool. A second non-MT scan was then collected using the same parameters without the MT pulse. Total scan time for both sets of images was 8 min.

### Image analysis

All analyses were performed by an experienced reviewer (MAH) blinded to clinical outcome. All images were visually evaluated for sufficient contrast between white and grey matter and motion or acquisition artifacts. If either image, with or without the MT pulse, was not of sufficient quality both were removed from the analyses. Of the 78 participants who underwent scanning at the 1-year time point, 76 sets were determined to have at least 1 image pair of sufficient quality for analysis.

**Figure 2:**
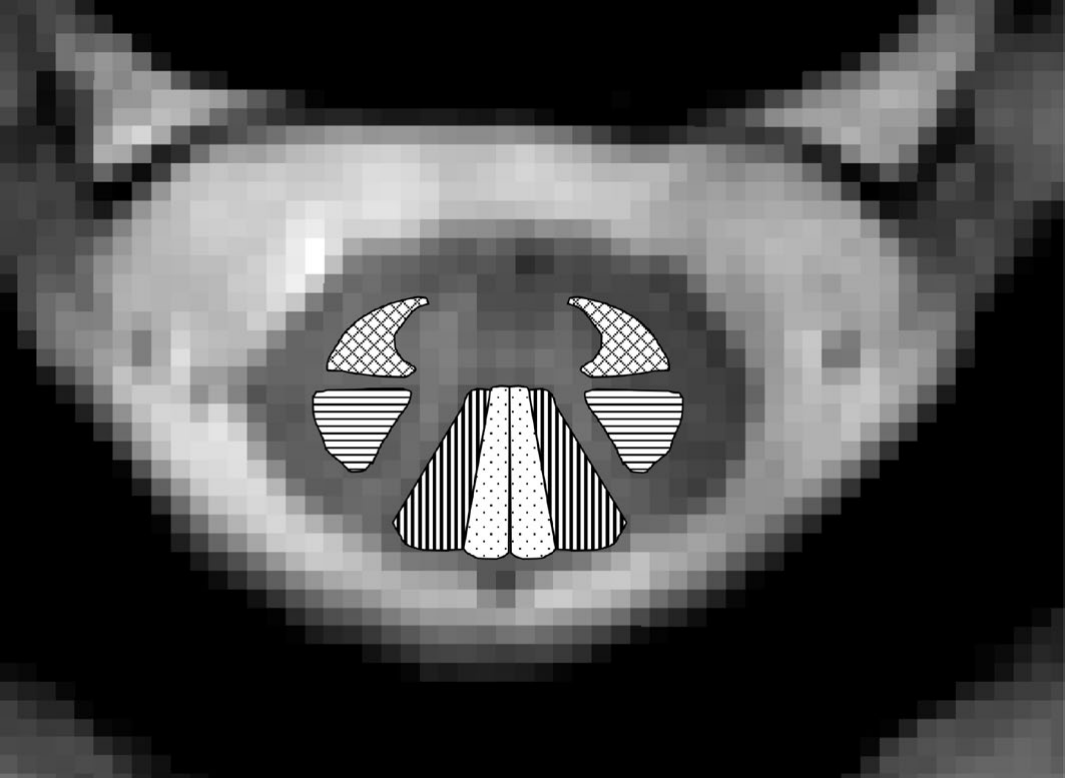
Example MR image from the C2/3 level, with highlighted bilateral large white matter tracts: corticospinal tracts (horizonal lines); cuneate fasciculi (vertical lines); gracile fasciculi (dotted); combined spinothalamic and spinoreticular tracts (crosshatched lines).

Individual tracts were analyzed using the open-source Spinal Cord Toolbox (SCT), version 3.2.7; a toolkit created specifically for spinal cord image processing and analysis^37^. Measurements were made from large motor and sensory white matter pathways: bilateral lateral corticospinal tracts (CST), combined spinothalamic and spinoreticular tracts (SPTH), and cuneate and gracile fasciculi (CF and GF respectively), illustrated in Fig. 2. The ascending spinothalamic and spinoreticular tracts are combined as necessitated by the atlas utilized in the SCT.

The image processing pipeline began by loading all anonymized images into a DICOM viewing database; then exporting and converting to the NIFTI file format for analysis. Binary masks of the spinal cord were first generated from the images with (I_MT_) and without the MT pulse (I_o_) using the sct_deepseg_sc function in the SCT; then manually corrected if necessary. The I_o_ image was then co-registered to the I_MT_ image in-plane first via the center of mass from the spinal cord masks and then by applying rigid transformations (translation and rotation) using a cross-correlation cost function, with a circular binary mask (diameter 35 voxels) centered at the spinal cord. Next, the PAM50 spinal cord template was registered to the mean of the co-registered I_MT_ and I_o_ images, first by centering on the spinal cord segmentation masks; then registered columnwise in the anterior and posterior directions^38^. The template to mean of I_o_ and I_MT_ registration was then fine-tuned via non-linear registration to the internal structures of the spinal cord using the white matter and grey matter masks generated by a deep learning convolution neural net (CNN), optimized for gray matter segmentation^39^. Registrations were visually inspected by a reviewer (MAH), blinded to clinical presentation, for accuracy. The magnetization transfer ratio (MTR) was calculated from the co-registered I_MT_ and I_o_ images with the following equation:

$${\text{MTR}} = { }\left( {\frac{{I_{o} - I_{MT} }}{{I_{o} }}} \right) \times 100\%$$

**Figure 3:**
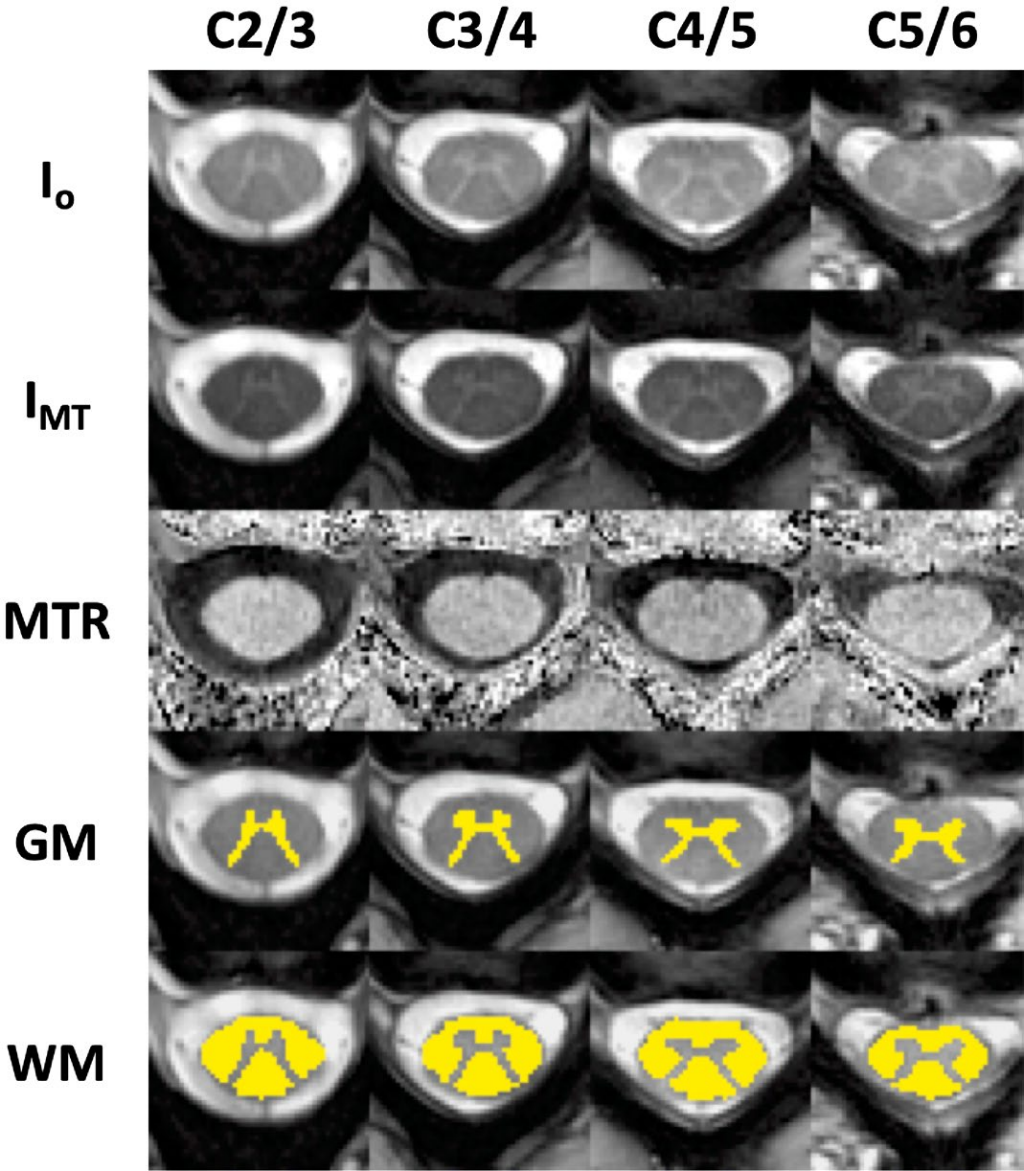
Example of co-registered images from C2/3, C3/4, C4/5, and C5/6 without and with magnetization transfer prepulse (respectively No MT and MT), magnetization transfer ratio (MTR) maps, and white (WM) and grey matter (GM) segmentations. MTR maps are shown scaled to minimum and maximum values within the spinal cord white matter.

MTR values of specific white matter tracts from the projections of the warped PAM50 atlas were then extracted using the maximum a posteriori method^40^. Example co-registered I_MT_ and I_o_ images, calculated MTR maps, and white and grey matter segmentations are displayed in Fig. 3.

Due to inherent differences in body composition and size of participants, differing inhomogeneities of the magnetic field within and between scans, and differences in transmitter and receiver coil loads, MT signals are difficult to reproduce on a participant-specific and slice-specific level^41,42^. A participant-invariant measure of the MT homogeneity, MTR*h*, was also calculated at each given slice, by taking the standard deviation of white matter MTR values for the specified tracts, and normalizing by the squared sum to remove any scale effects between participants or levels. This formulation is equivalent to calculations of fractional anisotropy in diffusion data. MTR*h* is calculated by the following equation:

$${\text{MTR}}h = \sqrt {\frac{{N_{A} }}{{\left( {N_{A} - 1} \right)}}} \sqrt {\frac{{\sum \left( {x_{A} - \overline{x}} \right)^{2} }}{{\sum \left( {x_{A} } \right)^{2} }}}$$

where $x_{A}$ represents the regional MTR values in the large white matter tracts of the cord, $\overline{x}$ is the average of the tract MTR values and *N*_*A*_ is determined by the number of tracts measured (8 in this study). The MTR*h* metric ranges from being completely homogenous, at 0, and increases as inhomogeneities in the measured quantities increase.

### Statistical analysis

Participant descriptive statistics (age and BMI) and NDI percentage scores were compared with ANOVA and Bonferroni-corrected post-hoc analyses, with significance level α = 0.05. MTR*h* analyses were split into 3 sections. The first analysis compared analysis of variance to test for differences in group means using clinical outcome as the only factor. A second analysis was triggered when significance was found in the first test case, comparing means using analysis of variance, but now including sex and clinical outcome as factors. For both analyses 1 and 2, the threshold for significance was α = 0.05. Post hoc analyses, correcting for multiple comparisons using the Bonferroni method, were performed if significance was found. Group MTR and MTR*h* means were calculated for each clinical outcome group and compared using JASP, an open-source statistics package^43^.

In the third section of the analysis, a linear mixed model was created for MTR and MTR*h* using least square means adjusted for BMI, age, and cervical level. In the linear mixed model, data excluded, due to low quality, was modeled using multiple imputation methods (5 sets). This analysis was performed in SASv9.4 (SAS Institute, Cary NC) using the functions: PROC MI, PROC MIXED, and PROC MIANALYZE. As part of this third analysis, correlation between NDI and all imaging measures (with imputation of excluded data) were calculated by Pearson’s product-moment correlation.

## Supplementary Information

Supplementary Information

## Data Availability

De-identified datasets used in this study are available from the corresponding author upon reasonable request.
