# Supplementary material for: Macromolecular changes in spinal cord white matter characterize whiplash outcome at 1-year post motor vehicle collision
Source: Sci Rep. 2020 Dec 17;10:22221. doi: 10.1038/s41598-020-79190-5 (PMC7747591; doi:10.1038/s41598-020-79190-5)
Supplement: Supplementary file 1 — Supplementary Information [file 41598_2020_79190_MOESM1_ESM.docx]

**Macromolecular changes in spinal cord white matter characterize whiplash outcome at 1-year post motor vehicle collision**

Mark A. Hoggarth^1,2^*, James M. Elliott^2,3^, Zachary A. Smith^4^, Monica Paliwal^4^, Mary J. Kwasny^5^, Marie Wasielewski^2^, Kenneth A. Weber II^6^, and Todd B Parrish^1,7^

1. Department of Biomedical Engineering, McCormick School of Engineering, Northwestern University, Evanston, IL, USA
2. Department of Physical Therapy and Human Movement Sciences, Feinberg School of Medicine, Northwestern University, Chicago, IL, USA
3. Northern Sydney Local Health District, The Kolling Research Institute and The Faculty of Health Sciences, The University of Sydney, St. Leonards, NSW, Australia
4. Department of Neurological Surgery, University of Oklahoma Health Sciences Center, Oklahoma City, OK, USA
5. Department of Preventive Medicine, Feinberg School of Medicine, Northwestern University, Chicago, IL, USA
6. Systems Neuroscience and Pain Lab, Department of Anesthesiology, Perioperative and Pain Medicine, Stanford University, Palo Alto, CA, USA
7. Department of Radiology, Northwestern University, Chicago, IL, USA

*Mark.Hoggarth@Northwestern.edu

| **Cervical Level** | **Recovered** | | | **Mild** | | | **Severe** | | |
| --- | --- | --- | --- | --- | --- | --- | --- | --- | --- |
|  | **Female** | **Male** | **Total** | **Female** | **Male** | **Total** | **Female** | **Male** | **Total** |
| *C2/3* | 16 | 12 | 28 | 20 | 4 | 24 | 10 | 3 | 13 |
| *C3/4* | 15 | 13 | 28 | 21 | 4 | 25 | 10 | 3 | 13 |
| *C4/5* | 15 | 11 | 26 | 19 | 4 | 23 | 9 | 3 | 12 |
| *C5/6* | 14 | 7 | 21 | 13 | 2 | 15 | 8 | 3 | 11 |
| *C6/7** | 8 | 6 | 14 | 8 | 0 | 8 | 7 | 3 | 10 |
| *C7/T1** | 9 | 6 | 15 | 7 | 0 | 7 | 3 | 2 | 5 |

**Supplementary Table S1**: Number of images with suitable contrast for analysis at each level for each clinical outcome group, by sex. C6/7 and C7/T1 were excluded from the statistical analysis due to low numbers of images with suitable contrast for imaging analysis.

|  | | | | | | | | |
| --- | --- | --- | --- | --- | --- | --- | --- | --- |
|  | **Comparison** | | **Mean Difference** | **SE** | **t** | | **Adj. p** |  |
| MTR*h* | RF | MF | -3.493e -4 | 0.008 | -0.045 | 1.000 | |  |
|  |  | **SF** | **-0.041** | **0.009** | **-4.38** | **< 0.001** | |  |
|  |  | RM | -0.006 | 0.009 | -0.624 | 1.000 | |  |
|  |  | MM | -0.021 | 0.013 | -1.604 | 1.000 | |  |
|  |  | SM | 0.021 | 0.014 | 1.457 | 1.000 | |  |
|  | MF | **SF** | **-0.041** | **0.009** | **-4.498** | **< 0.001** | |  |
|  |  | RM | -0.005 | 0.009 | -0.608 | 1.000 | |  |
|  |  | MM | -0.021 | 0.013 | -1.605 | 1.000 | |  |
|  |  | SM | 0.021 | 0.014 | 1.504 | 1.000 | |  |
|  | SF | **RM** | **0.035** | **0.01** | **3.527** | **0.008** | |  |
|  |  | MM | 0.02 | 0.014 | 1.4 | 1.000 | |  |
|  |  | **SM** | **0.062** | **0.015** | **4.143** | **< 0.001** | |  |
|  | RM | MM | -0.016 | 0.014 | -1.142 | 1.000 | |  |
|  |  | SM | 0.026 | 0.015 | 1.793 | 1.000 | |  |
|  | MM | SM | 0.042 | 0.018 | 2.381 | 0.271 | |  |
|  |  |  |  |  |  |  | |  |
| L SPTH | RF | MF | -1.896 | 0.941 | -2.015 | 0.676 | |  |
|  |  | **SF** | **-5.381** | **1.129** | **-4.767** | **< 0.001** | |  |
|  |  | RM | -3.169 | 1.079 | -2.937 | 0.055 | |  |
|  |  | MM | -0.024 | 1.603 | -0.015 | 1.000 | |  |
|  |  | SM | -1.903 | 1.708 | -1.114 | 1.000 | |  |
|  | MF | **SF** | **-3.485** | **1.09** | **-3.197** | **0.024** | |  |
|  |  | RM | -1.273 | 1.038 | -1.226 | 1.000 | |  |
|  |  | MM | 1.873 | 1.576 | 1.188 | 1.000 | |  |
|  |  | SM | -0.007 | 1.682 | -0.004 | 1.000 | |  |
|  | SF | RM | 2.212 | 1.211 | 1.826 | 1.000 | |  |
|  |  | **MM** | **5.358** | **1.695** | **3.162** | **0.027** | |  |
|  |  | SM | 3.478 | 1.794 | 1.939 | 0.807 | |  |
|  | RM | MM | 3.146 | 1.662 | 1.893 | 0.894 | |  |
|  |  | SM | 1.266 | 1.763 | 0.718 | 1.000 | |  |
|  | MM | SM | -1.88 | 2.125 | -0.885 | 1.000 | |  |

**Supplementary Table S2**: Post hoc comparisons with Bonferroni adjusted p-values for magnetization transfer ratio homogeneity (MTR*h*) and the magnetization transfer ratio of the left spinothalamic tract (L SPTH) between groupings with clinical outcome and sex as factors. Sex and clinical status are defined as: Recovered female (RF), Mild outcome female (MF), severe/moderate outcome female (SF), recovered male (RM), mild outcome male (MM), severe/moderate outcome male (SM).
